# Influence of a *Serratia marcescens* outbreak on the gut microbiota establishment process in low-weight preterm neonates

**DOI:** 10.1371/journal.pone.0216581

**Published:** 2019-05-21

**Authors:** Esperanza Escribano, Claudia Saralegui, Laura Moles, María Teresa Montes, Claudio Alba, Teresa Alarcón, Fernando Lázaro-Perona, Juan Miguel Rodríguez, Miguel Sáenz de Pipaón, Rosa del Campo

**Affiliations:** 1 Servicio de Neonatología, Hospital Universitario La Paz, and Universidad Autónoma de Madrid, Madrid, Spain; 2 Servicio de Microbiología y Parasitología, Hospital Universitario Ramón y Cajal, and Instituto Ramón y Cajal de Investigaciones Sanitarias (IRYCIS), Madrid, Spain; 3 Departamento de Bromatología, Facultad de Veterinaria Nutrición y Ciencia de los Alimentos, Universidad Complutense de Madrid, Madrid, Spain; 4 Servicio de Microbiología, Hospital Universitario La Princesa, and Universidad Autónoma de Madrid, Madrid, Spain; 5 Servicio de Microbiología, Hospital Universitario La Paz, Madrid, Spain; 6 Red de Salud Materno Infantil y del Desarrollo, Instituto de Salud Carlos III, Madrid, Spain; Children's Hospital Los Angeles, UNITED STATES

## Abstract

Adequate gut microbiota establishment is important for lifelong health. The aim was to sequentially analyze the gut microbiota establishment in low-birth-weight preterm neonates admitted to a single neonatal intensive care unit during their first 3 weeks of life, comparing two epidemiological scenarios. Seven control infants were recruited, and another 12 during a severe *S*. *marcescens* outbreak. Meconium and feces from days 7, 14, and 21 of life were collected. Gut microbiota composition was determined by 16S rDNA massive sequencing. Cultivable isolates were genotyped by pulsed-field gel electrophoresis, with four *S*. *marcescens* submitted for whole-genome sequencing. The expected bacterial ecosystem expansion after birth is delayed, possibly related to antibiotic exposure. The *Proteobacteria phylum* dominates, although with marked interindividual variability. The outbreak group considerably differed from the control group, with higher densities of *Escherichia coli* and *Serratia* to the detriment of *Enterococcus* and other *Firmicutes*. Curiously, obligate predators were only detected in meconium and at very low concentrations. Genotyping of cultivable bacteria demonstrated the high bacterial horizontal transmission rate that was confirmed with whole-genome sequencing for *S*. *marcescens*. Preterm infants admitted at NICU are initially colonized by homogeneous microbial communities, most of them from the nosocomial environment, which subsequently evolve according to the individual conditions. Our results demonstrate the hospital epidemiology pressure, particularly during outbreak situations, on the gut microbiota establishing process.

## Introduction

The relationship between our human cells and the microbial communities living inside us can be classified as mutualistic, commensal, or pathogenic. This consideration delimits the fine barrier that distinguishes colonization from infection, and can fluctuate over time due to influence from the host or from microbial and environmental factors. The influence of the intestinal microbiota on global human health has been confirmed [1–3], forming new research perspectives aimed to optimize the composition and the functionality of this bionetwork.

Adequate microbiota establishment in newborns is a process particularly relevant for their lifelong health [4], and its management represents a scientific challenge. Human bacterial colonization might start *in utero*, but the critical step begins with the exposure to maternal bacteria at birth and during the early postnatal period [5–6]. Bacterial populations fluctuate considerably during the first months of life, until a stable ecosystem is established when the infant is approximately 2–3 years of age [7–8]. Universal criteria defining a “normal” or “healthy” gut microbiota have not yet been established, which should be characterized by a high diversity, marked inter-individual variability, and conserved intraindividual stability. However, the composition of this ecosystem is influenced by numerous factors [9–10], such as the gestational age at which the neonate is born [11–12].

Preterm birth is the main cause of perinatal morbidity and mortality, as well as an important risk factor for death in the first 5 years of life [13]. A considerable increase in preterm birth rates over the past two decades has been reported worldwide, in both developed and developing countries [14]. In Spain, the preterm birth rate of all live births increased from 7.1% in 1996 to 8.2% in 2008, which is one of the highest rates in Europe [15]. This global tendency can be explained by several factors, including an early or advanced mother’s age, a small gap between pregnancies, low body mass index, multiple pregnancy, history of infectious diseases, stress, alcohol consumption, and periodontal disease [13]. Nevertheless, approximately half of the spontaneous preterm births have an unidentified cause, and it has been suggested that the composition of the maternal microbiota could play a relevant triggering role [7,16].

In low-birth-weight preterm infants (<2500 g), the gut microbiota composition and their biodiversity are aberrant, given the bacterial establishment is delayed by their prolonged hospital stay and their intense exposure to antimicrobials [12,17–19]. This fact explains, at least partially, why preterm infants have a very immature immune system and typically experience infectious complications [20–22]. In this context, *Serratia marcescens* is one of the most relevant emerging pathogens causing severe outbreaks in this population [23–24]. Pathogenic gut colonization during nosocomial outbreaks has frequently been reported; to our knowledge, however, the influence of an outbreak on the microbiota establishment process has not thus far been studied.

The aim of the present study was to sequentially analyze the gut microbiota establishment of low-birth-weight preterm neonates admitted to a single neonatal intensive care unit (NICU) during their first 3 weeks of life, comparing two epidemiological scenarios: a normal period and a period with a nosocomial *S*. *marcescens* outbreak.

## Materials and methods

### Preterm neonate inclusion criteria and sampling procedure

La Paz University Hospital (Madrid, Spain) has a 23-bed level III NICU, from which 19 low-birth-weight preterm neonates (<32 weeks gestational age) were recruited in two separate periods: (A) during an epidemiologically normal period in 2015 (control group, n = 7); and (B) during a severe *S*. *marcescens* outbreak from December 2016 to March 2017 (outbreak group, n = 12). Despite the different sampling periods, there were no significant changes in the NICU. It is important to note the data lack in the control group about antibiotic consumption, and clinical data, as a limitation of our work. From each preterm infant, four fecal samples were collected after birth: meconium, and feces from 7, 14, and 21 days of life. Although our initial aim was to extend the recruitment period, logistical limitations limited the study to the first three weeks. The samples were directly recovered from the diaper using a sterile plastic stick and immediately stored at -80°C. Although our intention was to collect fecal samples immediately after deposition, we cannot rule out the possible contact and contamination with urine. However, the contribution of the urinary microbiota should be insignificant.

For the control group, only DNA from fecal samples was available, whereas for the outbreak group bacterial growth was obtained by culture-dependent techniques in addition to DNA. The ethics committee “Comité Ético de Investigación Clínica del Hospital Universiatrio La Paz” approved the study (reference HULP3551), and the data of all the neonates were obtained from their clinical chart. The infants were categorized according to four variables: 1) epidemiological situation (normal or *S*. *marcescens* outbreak); 2) delivery mode (vaginal or C-section); 3) gestational age (extremely preterm: <28 weeks; very preterm: 28–30 weeks; or moderately preterm: 30–32 weeks); and 4) birth weight (<1000 g; 1000–1500 g; or >1500 g).

### Sample processing

Fecal samples from the *S*. *marcescens* outbreak group were slowly defrosted at -20°C for 24 h and 4°C for another 24 h, in order to avoid bacterial death. Portions between 0.3–0.5 g of each sample were inoculated into Brain Heart Infusion (BHI) broth (Difco, Detroit, Michigan) and incubated at 37°C for 24 h as a bacterial pre-enrichment. Cultivable bacteria were isolated in selective and nonselective agar media from the BHI tube, including agar plates of M-*Enterococcus*; De Man, Rogosa and Sharpe (MRS); mannitol salt; McConkey; and Columbia, with 5% sheep blood. The culture media were purchased from Difco, and the plates were incubated at 37°C for 24–48 h, including 5% CO_2_ for the blood agar plates, and anaerobic conditions for the MRS plates. Colony identification was performed by matrix-assisted laser desorption ionization time-of-flight mass spectrometry (Bruker, Germany), and all the isolates were conserved at -80°C in semi-skimmed milk. In parallel to bacterial cultures, total DNA was obtained from fecal aliquots of 0.3–0.5 g with the QiaAMP kit (Qiagen, Germany), determining their concentration and quality by Qubit fluorometer (Invitrogen, USA).

### 16S rDNA next-generation sequencing

The fecal DNA samples were sent to FISABIO (Valencia, Spain) for massive Mi-Seq 2 × 300 bp paired-end Illumina 16S rDNA sequencing (Cod. 15044223 Rev. A) from the V3 and V4 regions, which were amplified with the following primers: (Forward Primer: 5’-TCGTCGGCAG CGTCAGATGTGTATAAGAGACAGCCTACGGGNGGCWGCAG), (Reverse Primer: 5’-GTCTCGTGGGCTCGGAGATGTGTATAAGAGACAGGACTACHVG GGTATCTAATCC) [25]. Shannon and Chao1 indexes were used for alpha bacterial diversity estimation and were calculated, eliminating taxa with fewer than three lectures. Taxonomic affiliations were assigned using the Ribosomal Database Project (RDP) classifier, and reads with an RDP score below 0.8 were assigned to the upper taxonomic rank, leaving the last rank as unidentified. Sequence quality was measured according to the following parameters: minimum length, 250 bp; trimming quality measure type, mean; trimming quality number from 3’ extreme, 30; trimming quality window, 10 bp. Relative abundance and contingency tables included singletons and very low-represented taxons. The statistical analysis was performed using R statistical software and several open source libraries. The quantitative data of the reads were homogenized using their relative percentage from the total reads of each sample to facilitate the comparison between samples. Finally, the Galaxy Huttenhower Platform (http://huttenhower.sph.harvard.edu/galaxy) was used in order to calculate the Linear Discriminant Effect Size Analysis (LEfSe) algorithm and to obtain cladograms in which microbial taxa that explain significant differences among groups of samples were represented. A free software platform was used according to paper instructions [26]. For the statistical analysis, samples with fewer than 1,000 reads were dismissed, and all the samples from patient 7B were excluded due to a lack of some demographic and perinatal information. Fasta files are deposited in the NCBI web site under the Bioproject PRJNA510235 reference.

### Pulse-field gel electrophoresis typing

Cultivable bacterial isolates were genotyped by pulse-field gel electrophoresis (PFGE), using the habitual particular settings for the PFGE protocol and also for the restriction enzymes (*Sma*I for staphylococci and enterococci, *Xba*I for *Escherichia coli*, and finally *Spe*I for *Serratia* and *Klebsiella*). The PFGE pattern analysis was made with Phoretix 5.0 software (TotalLab, Newcastle upon Tyne, UK), and the representation of the results was made based on Dice coefficients and the unweighted pair group method with arithmetic mean algorithm.

### Whole genome sequencing

Four *S*. *marcescens* isolates from different infants were submitted to whole genome sequencing (WGS) by MiSeq technology (Illumina), and the genetic relationships were analyzed in the Galaxy Huttenhower Platform. The genome sequences are deposited in the European Nucleotide Archive database with the accession numbers QYRU00000000 and QYSA00000000 for Clone A, and QYRV00000000 and QYSB00000000 for Clone B.

### Fungi identification

The internal transcribed spacer (ITS)-1 region was amplified from the total fecal DNA using polymerase chain reaction (PCR) in order to analyze fungi diversity, using the primers ITS1-F (5’-CGCCCGCCGCGCGCGGCGGGCGG GGCGGGGGCACGGGGGGCTTGGTCATTTAGAGGAAGTAA-3’) and ITS1-R (5’-TCCTCCGCTTATTGATATGC-3’) [27]. Afterward, the amplicons were separated with denaturing gradient gel electrophoresis (DGGE), using the D-CODE system (BioRad Laboratories, USA). The gel bands were cut, reamplified, and sequenced in an AQBI Prism 7000 apparatus.

### Statistical analysis

The Kruskal-Wallis index was used for differences between two groups of samples (comparing medians) for each variable of study, and Dunn’s test was used when more than two groups were defined regarding a variable of study. The principal component analysis was applied for the multivariant analysis regarding taxonomical data in order to see differences between groups according to the variables of study. Statistical significance was adjusted to *p* < .005.

## Results

### Characteristics of the preterm infants

The demographic and clinical characteristics of both newborn groups are shown in Table 1 and in S1 Dataset. The most relevant differences were that the control infants presented a higher weight at birth and a lower incidence of sepsis. One neonate from the control group (14.3%) suffered from early-onset sepsis, whereas late-onset sepsis was microbiologically or clinically diagnosed in five neonates from the outbreak group (41.7%), being *Staphylococcus epidermidis* and *S*. *marcescens* the microorganisms implicated. Data on antimicrobial therapy were only available for the outbreak group and were included the administration of prophylactic cefazolin during labor (10 infants), empiric treatment based on ampicillin, gentamicin, and clarithromycin during the first week of life (8 infants), and treatment that included vancomycin (7 infants), cefotaxime (2 infants) piperacillin/tazobactam (2 infants), meropenem (1 infant), and amikacin (3 infants) for the second and third weeks of life.

**Table 1:** Clinical and demographic characteristics of the preterm infants of both groups.

Characteristic	Control Group (7 infants)	Outbreak Group (12 infants)	*p* value
Weight at birth (g)	1462 (720–1890)^a^	971 (600–1537)^a^	0.009
Gestational Age (weeks)	30 (25–31)^a^	28 (25–31)^a^	0.26
Vaginal delivery (n, %)	5, 71.4%	3, 25%	0.04
Male sex (n, %)	5, 71.4%	2, 16.6%	0.0001
Sepsis (n, %)	1, 14.3%	6, 50%	0.04
Length of stay (days)	14 (5–140)^a^	55 (7–89)^a^	0.1

^a^Values expressed as the median value and the range (between parentheses).

### Gut microbiota establishment by next-generation sequencing

**Fig 1:**
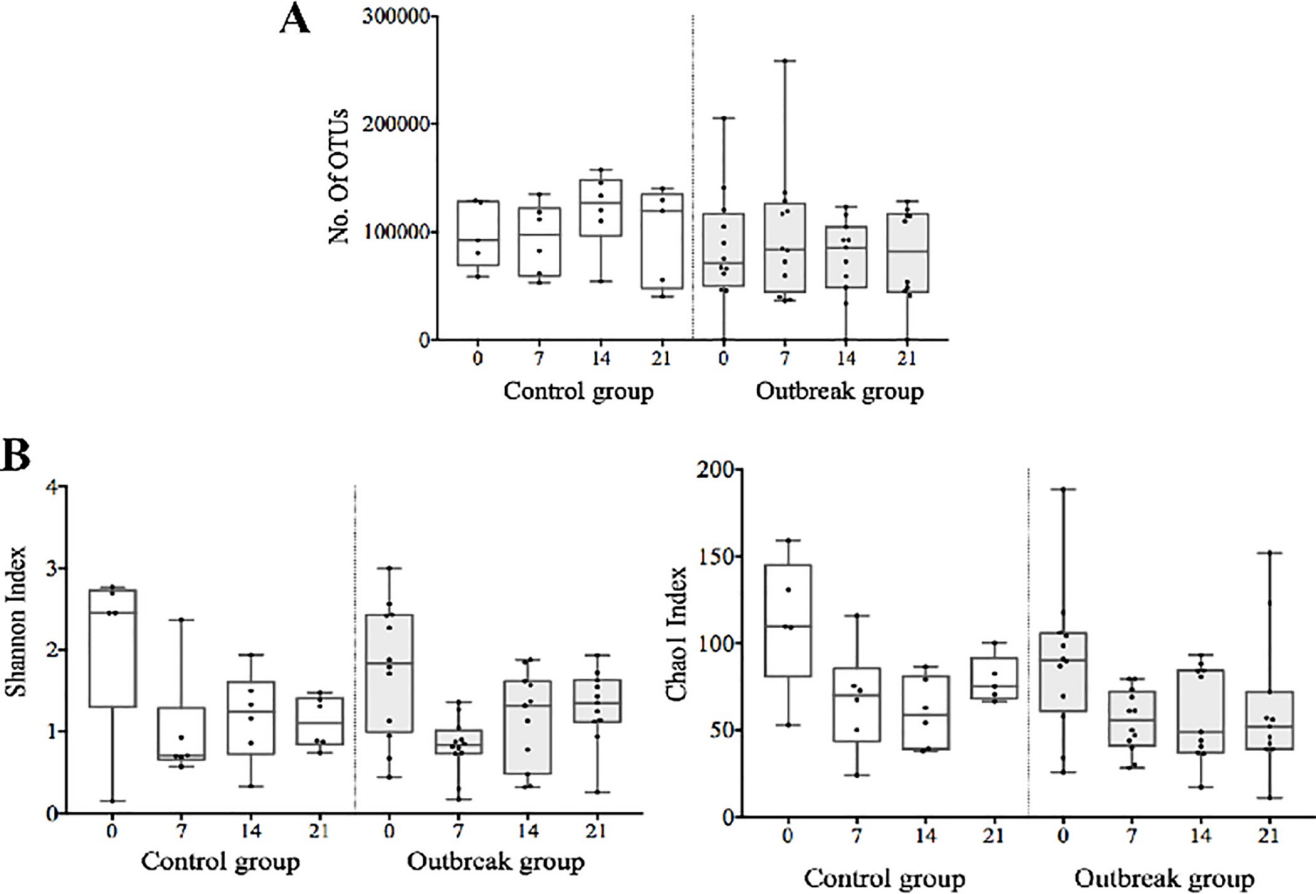
Number of operational taxonomic units (OTUs) **(A)**, and alpha diversity measured by the Chao1 index **(B)** in all samples studied.

The number of operational taxonomic units (OTUs) and the alpha diversity indexes of the meconium samples were similar to the further fecal samples (Fig 1), indicating a delayed bacterial establishment process. LEfSe analysis allowed us to explore the differences in microbiota composition between the control and the outbreak groups for the four variables stated above (epidemiological situation, delivery mode, gestational age, and birth weight).

**Fig 2:**
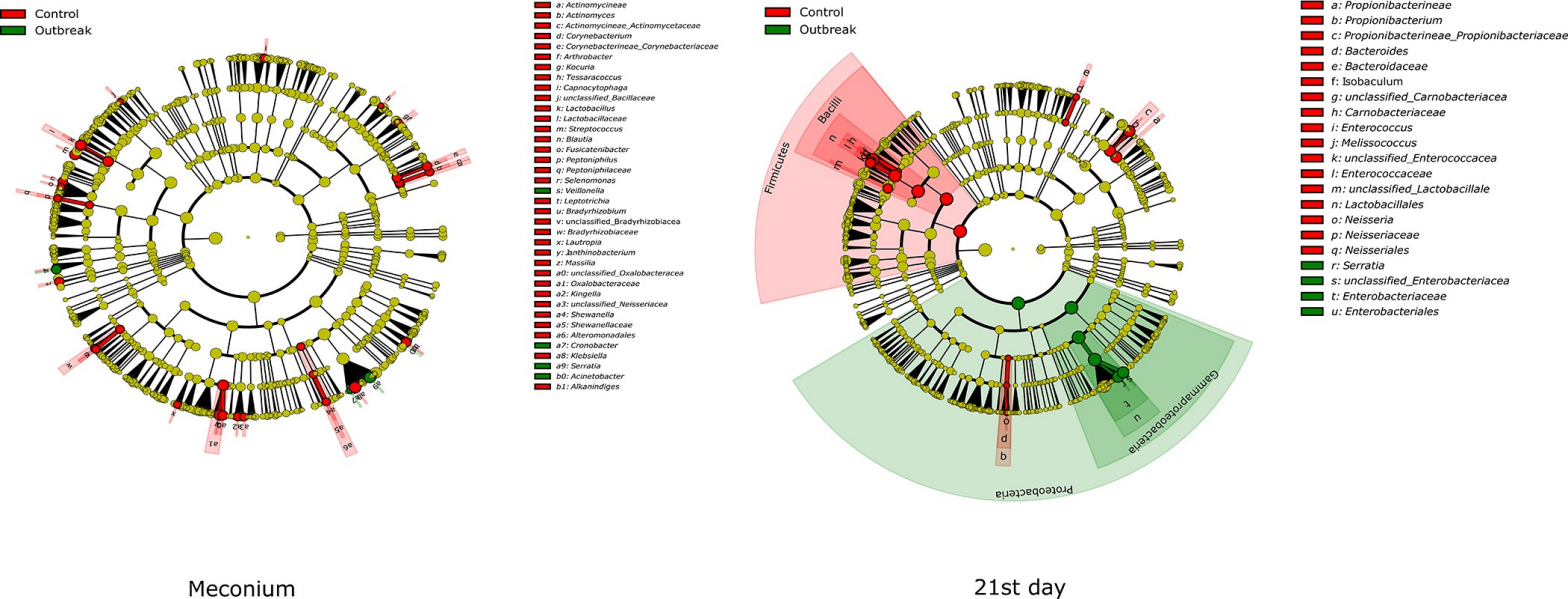
Cladograms showing the significant differences of gut microbiota composition in meconium and 21 days feces between control and outbreak groups.

Significant differences in the gut microbiota composition of the control and outbreak groups were detected in meconium and in the day 21 samples and were more relevant in those found at day 21 (*p* = .0024 at the genus level and *p* = .073 at the *phylum* level) (Fig 2). The outbreak group was characterized by a higher proportion of γ-*Proteobacteria*, related to a higher density of *Serratia*, and with lower proportions of the *Firmicutes* and *Fusobacteria phyla*.

**Fig 3:**
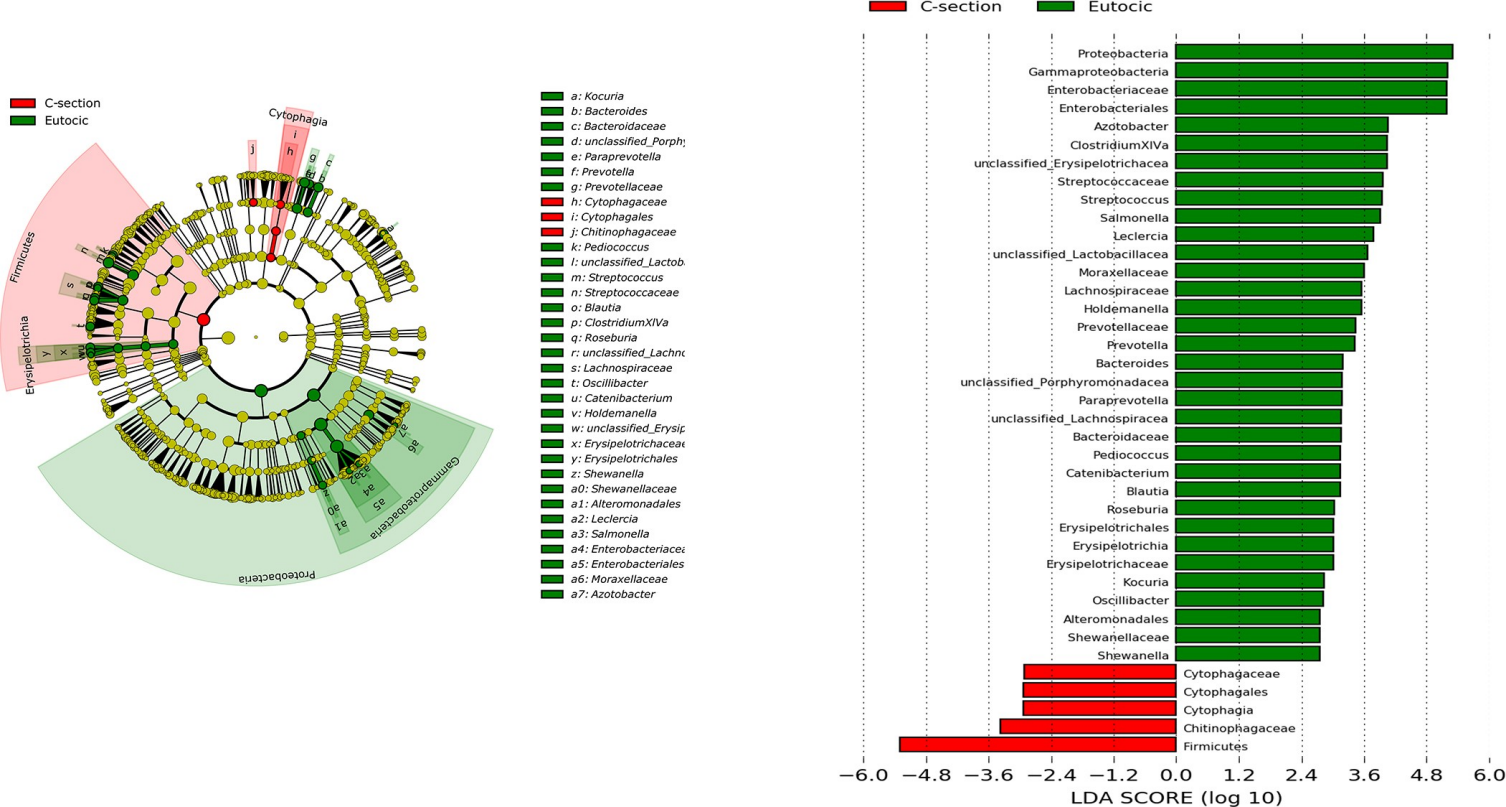
Significant differences in the gut microbiota of meconium by the delivery mode.

In relation to the delivery mode, differences between vaginal delivery and C-section delivery were only significantly different at day 0 (*p* = .0066 at the genus level and *p* = .0363 at the *phylum* level) (Fig 3). Significant differences among bacterial communities regarding gestational age and birth weight were not detected.

**Fig 4:**
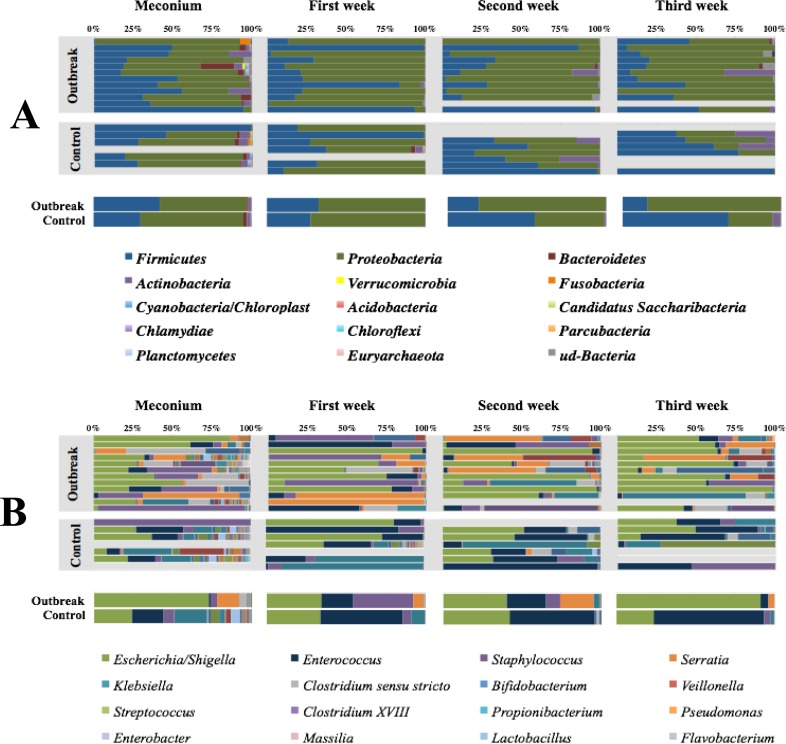
**(A)** *Phyla* percentage in each sample and infant, and summary of both groups expressed as the medians values. **(B).** Genera percentage in each sample and infant, and summary of both groups expressed as the median values. The 16 most abundant genera are highlighted in the figure, although up to 215 genera were detected in the samples analyzed in this study.

Taking into account all 55 samples, the predominant *phylum* during the first weeks of life of the low-weight preterm infants was *Proteobacteria* (median±SD 70.1% ± 26.9%, range 0.3%–99.4%), followed by *Firmicutes* (median±SD 22.1% ± 26.8%; range 0.05–99.4); and although up to another 23 *phyla* were detected, their contribution was nearly anecdotic (Fig 4A). At the genus level, the abundance of *Escherichia/Shigella sp*. increased over the studied period while that of *Enterococcus sp*. and *Staphylococcus sp*. decreased (Fig 4B).

Reads accounting for the predator bacteria *Bdellovibrio* (2 infants), *Peredibacter* (1 infant), and *Vampirovibrio* (1 infant) were found in meconium samples from the outbreak (3 infants) and the control (1 infant) groups. The relative abundance of predatory species was extremely low (0.004%–0.11%), and none could be detected in the subsequent fecal samples.

*Serratia* was detected in the meconium samples from all the patients in the outbreak group (median value, 5558 OTUs), whereas this genus was considerably less abundant among the meconium samples from the control group (median value, 180 OTUs) (Table 2). Also noticeable was that the *Serratia* reads considerably increased just before the diagnosis of *Serratia* sepsis in some patients from the outbreak group, and particularly in infant O11, who finally died from *S*. *marcescens* sepsis at day 10 after birth. The gut enrichment of *Serratia* sequences in this infant was manifest, reaching levels of 95% of the total intestinal microbiota at day 7 (Table 2).

**Table 2:** *Serratia* abundance detected by molecular tools and distribution of the two major clones detected in the outbreak group. The underlined isolates were submitted to whole genome sequencing. High abundance of *Serratia* by NGS is marked in light grey color, whereas the dark grey means a clear dominance of the *Serratia* genera.

	INFANT	Meconium		Day 7		Day 14		Day 21	
		Cultivable	NGS (%)	Cultivable	NGS (%)	Cultivable	NGS (%)	Cultivable	NGS (%)
**OUTBREAK** **GROUP**	O1	**Clone A**	5.4		0.002		12.1		1.0
	O2		12.4		0.003		0.2		1.7
	O3		2.5	Clone B	17.3		19.0		
	O4		2.4	Clone B	2.4	Clone B	4.8		16.9
	O5		1.8		0.003		0.04	Clone A	28.8
	O6		11.8	Clone B	5.1	Clone B	26.5	Clone B	37.4
	O7		2.9	**Clone B**	0.2		35.5		4.9
	O8		1.9		0.7		1.3		0.02
	O9		2.0		0.004		0.004		0.006
	O10	**Clone A**	59.5	Clone A	78.5	Clone A	0.8	Clone A	0.4
	O11		3.1	**Clone B**	94.5				
	O12		0.3	Clone A	1.3		0.1		1.2
**CONTROL GROUP**	C1		0.004		0.005				0.008
	C2		0.006		0.001		0.001		0.006
	C3		0.1		0.0007		0		0
	C4				0.06		0.2		0.01
	C5		0.8				3.1		
	C6		0.06		0		0		
	C7				0.0008		0		0.002

### Characterization of cultivable isolates from the outbreak group

**Fig 5:**
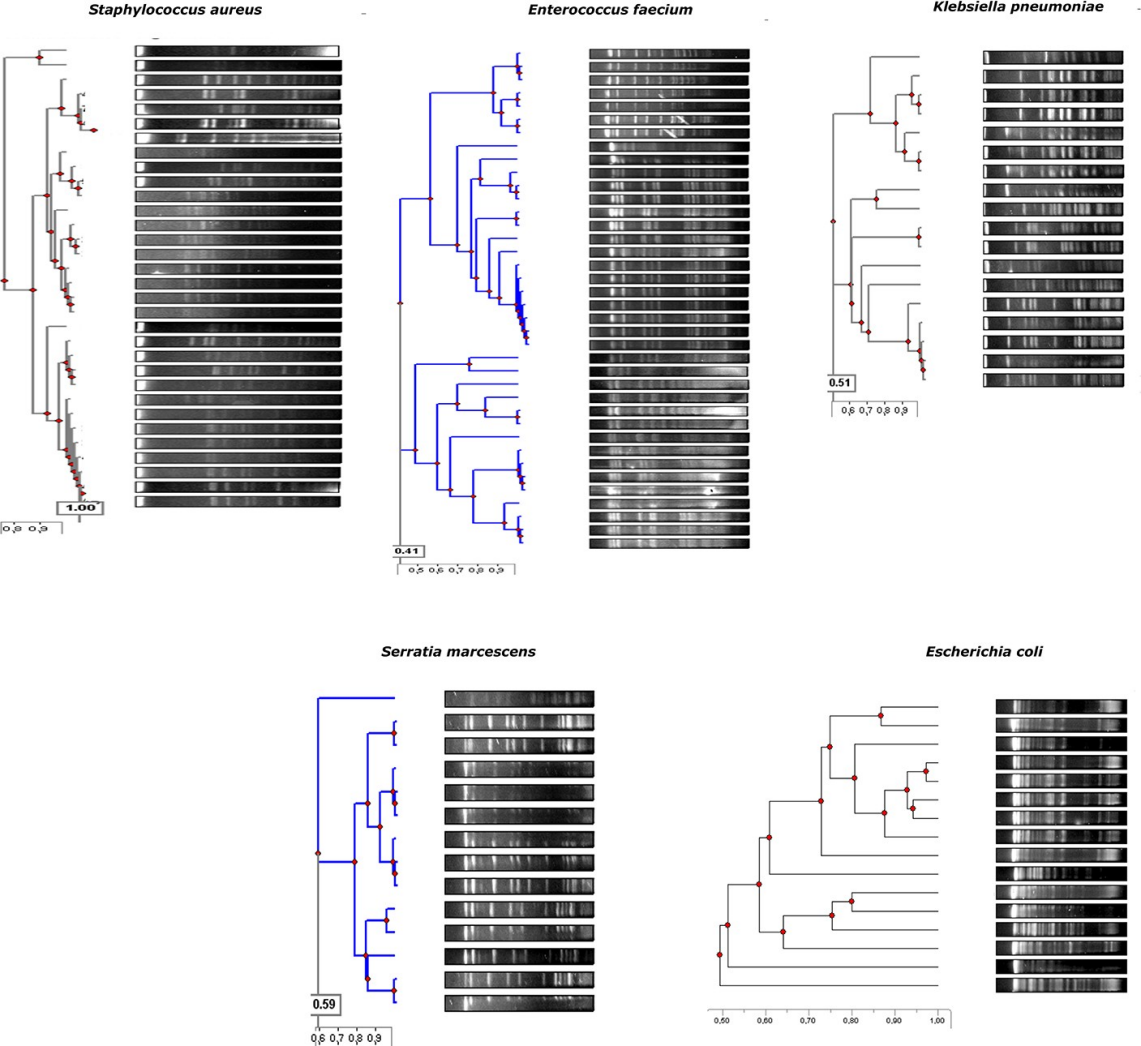
Dendrograme showing the genetic relationship among the cultivable isolates based on the Dice’s coefficient.

A total of 16 *E*. *coli* (8 infants), 35 *Enterococcus faecalis* (11 infants), 2 *Enterococcus faecium* (1 infant), 12 *Klebsiella oxytoca* (7 infants), 6 *Klebsiella pneumoniae* (5 infants), 32 *S*. *epidermidis* (all 12 infants), 1 *Serratia liquefaciens* and 14 *S*. *marcescens* (8 infants) isolates were recovered. Regarding the meconium samples, the cultivable microorganisms were *S*. *epidermidis* (7 infants), *E*. *faecalis* (3 infants), and *S*. *marcescens* (2 infants). Only four meconium samples (33.3%) did not yield viable microorganisms. PFGE analysis showed that isolates recovered from different samples from the same infant were identical or closely related (Fig 5).

Similarly, a single *E*. *faecalis* pulsotype was detected, colonizing three infants and two major clones of *K*. *pneumoniae* in two infants. Among the *Serratia* isolates, two genetically unrelated clones were detected, affecting four and five infants each. The relative abundance of *Serratia* in the gut microbiota of each infant is presented in Table 2, which compares both next-generation sequencing (NGS) and microbiological culture techniques. It shows the high counts of *Serratia* in all samples from the outbreak group, confirming the results previously observed in the NGS analysis.

### WGS of *Serratia* clones

**Fig 6:**
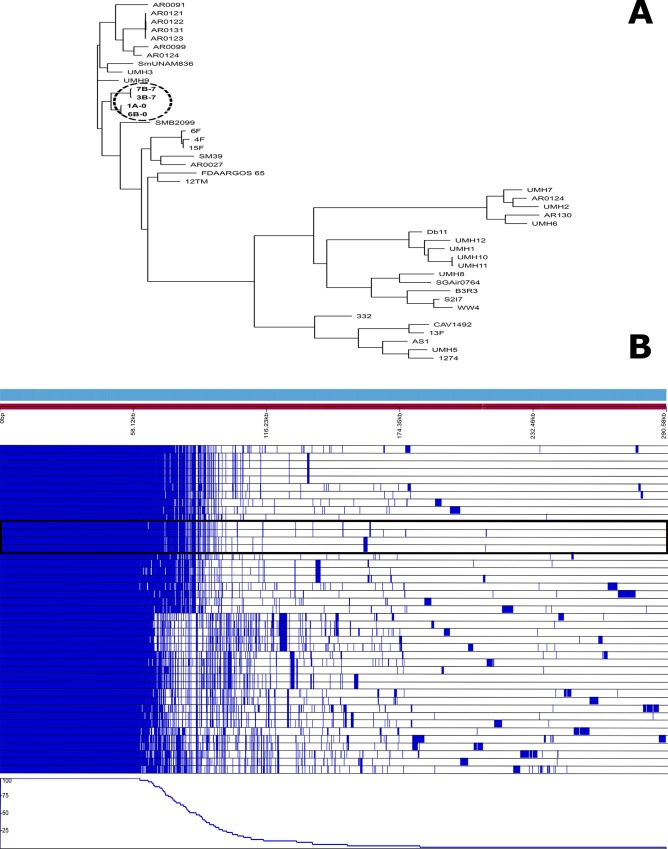
Phylogenic representation of the 4 *S*.*marcescens* genomes sequenced in this study and comparison with other 32 public *S*. *marcescens* genomes **(A).** The second part of the figure **(B)** represents the common core of all 36 genomes versus the isolate-specific genes. Our 4 isolates grouped together in both analysis and are marked.

Four *Serratia* isolates representing the two dominant PFGE clones associated with sepsis in the outbreak group were submitted to WGS (Fig 6). The genome analysis confirmed two phylogenic linages unrelated to others previously published. It is important to note that the isolate causing the sepsis and death of infant O11 was identical to the isolate obtained from infant O7, who had a satisfactory clinical evolution. WGS allowed the characterization of the resistome (*aac*(6’)-*Ic*-1, *bla*_SRT-2-1_, *bla*_SRT-1-1_, and *qnr*E) and the virulome (*che*B, *che*R, *che*W, *che*Y, *flg*B, *flg*C, *flg*G, *flg*I, *flh*A, *flh*C, *flh*D, *fli*A, *fli*G, *fli*I, *fli*M, *fli*N, *fli*P, *fli*Q, and *fli*Z), which was uniform in all four isolates. Nevertheless, our isolates remained susceptible to most antibiotics, given the resistant genes detected were located on the chromosome and not transferable.

### Fungi detection

PCR-DGGE yielded positive amplifications in infant 6 (days 21 and 28) and infant 8 (days 14 and 21), and the nucleotide sequences of these amplicons corresponded to *Candida albicans*.

## Discussion

In the present study, we have described the succession of the gut microbiota composition from meconium to the first 3 weeks of life of low-weight preterm infants, comparing two epidemiological scenarios from the same NICU. The primary result of our work is related to the *S*. *marcensens* outbreak influence on infant gut microbiota patterns, including meconium, which might have been modified even before birth in relation to the hospital admission of mothers to prevent premature birth. Although there is marked inter-individual variability, the gut microbiota of low-weight premature infants is dominated by *Proteobacteria phylum*, particularly *E*. *coli*. Moreover, the expected bacterial ecosystem expansion after birth appears to be delayed, probably in relation to antibiotic exposition. In that sense it is important to remark the data lack about antibiotic prescription on the control group, that represent an important limitation of our work.

Regarding to the outbreak group, all but two infants received empiric antibiotics immediately after birth, and most required culture-guided antibiotic treatments during their admittance, including up to three different antimicrobial families. However, the high incidence of infectious complications among preterm neonates is the main argument used to justify the wide empiric and culture-guided use of antimicrobials in preterm infants, particularly in those with low birth weight, as in our case. Early biomarkers of preterm sepsis, together with the development of microbiome-based approaches, are urgently required to reduce antibiotic use in NICUs [28–29].

Prematurity is the main cause of neonatal morbidity and mortality, and the establishment of an adequate gut microbiota appears to be one of the most promising strategies to improve preterm infants’ health and to reduce the impact of sequelae later in life [30–31]. Preterm infants admitted to an NICU have a high risk of infection, and *S*. *marcescens* is one of the most relevant nosocomial pathogens;^23^ their intestinal carriage has been identified as a potential reservoir [32].

Our results demonstrated considerable *Proteobacteria* enrichment in both preterm infant groups, although the enrichment was significantly higher in the outbreak group from the second week of life, in concordance with other authors [33–36]. Previous studies found that *Proteobacteria* (mostly *Enterobacter* and *Photorhabdus*), *Firmicutes* (mostly *Enterococcus* and *Lactobacillus*), and *Actinobacteria* (*Bifidobacterium*) dominated the microbial composition of meconium [37]. *E*. *coli*, *Staphylococcus sp*., *Klebsiella sp*., and a high rate of facultative anaerobes also commonly appear in the meconium of preterm neonates [38–39]. Our study demonstrated significant differences between the control group dominated by *E*. *coli* and *Enterococcus* and the outbreak group with higher densities of *E*. *coli* and *Serratia*. Recent data have shown that current 16S rDNA technology is not applicable for the gut ecosystem of premature infants [40]; this is an important limitation of our work, probably showing lower detection of the *Bifidobacterium* population. A recent work also demonstrated that *Bifidobacterium* density is related to the gestational age [12].

Curiously, the meconium samples from both groups were characterized by high interindividual variability and similar alpha diversity as the subsequent fecal samples, pointing to a marked delay in the establishment of the ecosystem. Similar results have been reported in very-low-birth-weight preterm infants [17]. Other authors have described an increase of fecal microbiota complexity during the NICU stay [41].

Although intrauterine fetal gut colonization is still a controversial issue [6–8], the detection of the *Serratia* microorganism in meconium samples suggests the possibility that its preterm colonization could start before birth. Most of the mothers in our study (7 of 12) were admitted to prevent a premature birth by various causes for a median period of 9 days before birth, with a range from 1 to 49 days. In contrast, the mother whose infant died from *S*. *marcescens* sepsis was admitted only on the day before delivery. Whereas this microorganism was scarcely represented in the meconium, it was very dominant at day 7, a fact that probably preceded the blood translocation and the sepsis episode that occurred at day 10. Therefore, a systematic routine exploration for potential enrichment of specific gut bacterial populations could possibly contribute to prevention of bacteremia in susceptible groups of patients, such as preterm infants. Recently, a novel functional methodology using volatile organic compounds as biomarkers for early detection of gut bacterial enrichment was reported [42]. Our results also demonstrated that some epidemic microorganisms, such as *S*. *marcescens*, are able to colonize and eventually infect preterm neonates even when state-of-the-art preventive measures have been applied. The PFGE analysis grouped isolates colonizing the 12 infants in the outbreak group into two major clones, whereas the WGS revealed a close relationship between them, suggesting the existence of a common ancestor. These molecular techniques also revealed that the virulome of the strain causing bacteremia and death was identical to other strains with clinical successful evolution, reinforcing the hypothesis that the unclear barrier delimiting colonization from infection is influenced by numerous factors.

Some OTUs assigned to the mandatory predator bacteria *Bdellovibrio*, *Vampirovibrio*, and *Peredibacter* were detected in the three meconium samples analyzed in this study. Such bacteria need to predate other bacteria to grow and reproduce and are considered to be important ecological balancers of the microbial communities [43]. Few studies have focused on these bacteria in human ecosystems; however, their presence in meconium samples suggests that they might not be infrequent in the gut microbiota. A predator’s inoculation could represent an ecological tool to modulate bacterial communities, taking into account predator-prey specificity [44].

The preterm nutrition policy of the hospital specifies neonates be fed maternal milk, although this is typically combined with human milk from donors and with preterm-adapted formulas. All the participating preterm infants received all three types of milk during the study, and although such data are not detailed, we are aware that this factor also influences the gut microbiota establishment. Maternal milk can reshape the infant gut microbiota [9], contributing its own site-specific microbiota [45–47], but also promoting the increase of a precise population by its prebiotic action [48]. Thus, it would be suitable to include in the microbiota profiling scheme the differentiation between living and dead bacteria in order to identify real colonizers from casual bacterial passengers associated with food intake [49].

Globally, our results indicate that, regardless of their perinatal settings, preterm neonates admitted to the same NICU are initially colonized by similar microbial communities that later evolve according to individual conditions. A *Serratia* outbreak influence on the establishment of the gut microbiota appears to be universal from the first days of admission; however, our results might also be applied to outbreaks caused by other microorganisms. This highlights the importance of the environment regarding the pattern of gut colonization of hospitalized preterm infants.

## Supporting information

S1 Dataset Supplementary clinical data. (XLSX)
